# Policy-Development and Deference to Moral Experts

**DOI:** 10.1007/s11158-022-09577-w

**Published:** 2023-02-09

**Authors:** Jakob Elster

**Affiliations:** Norwegian Centre for Human Rights, University of Oslo, Oslo, Norway

**Keywords:** Moral experts, Policy, Moral deference, Public justification, Democratic legitimacy

## Abstract

The involvement of ethicists, philosophers or others who might qualify as ‘moral experts’ in policy-development, where they are sometimes, typically as members of a committee, given an advisory role, is often seen as problematic, for several reasons. First, there may be doubts as to the very existence of moral experts, and it may be hard to know who the moral experts are. Next, even if these problems are solved, giving experts a special role in policy-making might be problematic from a democratic point of view, if it involves politicians deferring to the moral judgements of experts. The paper considers possible replies to this problem of moral deference. One reply is that moral deference is unnecessary, because even moral non-experts are well equipped to assess the arguments offered by moral experts; I argue that this reply underestimates the complexity of moral arguments. Another reply is that if moral experts are simply given the ‘technical’ role of clarifying which concrete positions that follow from the values which decision-makers already accept, deference is not problematic. I will argue that this reply underestimates how a given set of moral values underdetermines which concrete positions follow from it. Finally, I will consider and defend the reply that since policy decisions are subject to a requirement that they be justified within the limits of public reason, and since these limits include a requirement that the justification be accessible, moral experts are barred from providing policy advice which rests on too complex moral arguments.

## Introduction

Political decision-makers, whether they are lawmakers in parliament or members of the executive, often rely on the advice of experts before making decisions. In order for a policy decision to be sound, it must normally build on factual knowledge, and in complex matters politicians cannot be expected to have, nor have the time to gather, the required knowledge themselves. Thus, politicians rely on experts to inform them about a range of issues, from climate change to health interventions. The increasing and inescapable use of experts in policy processes, which Holst and Molander name ‘the fact of expertise’ (Holst and Molander 2017), has led some to worry about a decrease in the democratic legitimacy of policy decision, as there seems to be a tension between ideals of democratic equality and privileged roles given to experts (for discussions, see e.g. Moore 2017, ch.2 and Lafont 2020, ch. 3.1).

At the same time, there are good reasons to allow experts to play a role in the policy process. First, it is hard to see how politicians can do without input from experts on complex empirical questions, as a policy decision is likely to be sub-optimal if it builds on false or inaccurate factual premises. Next, one might argue that as long as the experts only pronounce themselves on factual matters, such as the probability that a given measure will reduce the spread of a virus, the experts do not really encroach on the proper domain of politics. The reason is that from a set of factual premises, it never follows directly what one ought to do or which policy ought to be adopted. In order to reach an actual policy decision, we must also have normative premises, and as long as it is the politicians, and not experts, who provide these normative premises, there is no particular problem pertaining to the legitimacy of the policy decision, even if the factual premises of the decision were provided by experts.^1^

Based on this line of thought, we can formulate the *standard view* on the role of experts in policy development as follows: it is legitimate that politicians build on *technical expertise*—i.e. expertise about factual matters—when identifying the descriptive premises for their policy decisions, but it is not the proper role of experts to give advice as to the normative premises of a decision.^2^ This view can be illustrated with a dilemma which faced many countries during the COVID-19 pandemic starting in 2020: whether to keep schools closed to curb the spread of COVID-19. According to the standard view, politicians can rely on technical experts providing input as to the various costs (for example in terms of children’s mental health) and benefits (notably in terms of decreasing contagion) of keeping schools closed. But politicians should not, on this view, rely on expert advice as to how to weigh these costs and benefits against each other, as this is a normative question. A motivation for the standard view is that unlike factual matters, which are normally not the subject of democratic decisions, normative questions are at the core of democratic decision-making and allowing experts a role in this domain would be problematic in a way which allowing technical experts a role is not. Since politicians do not vote over whether closing schools would curb the spread of a virus, experts may give their input on this factual question without encroaching on the domain of politics; but since politicians do vote over whether schools should be closed (given the factual supposition that this would curb the spread of the virus), experts should refrain from giving input on this further question.

This standard view is, however, challenged by the use of *moral* experts as part of the political decision-making process. The use of moral expertise, often in the form of ethics commissions, can be found across a variety of policy domains, but is perhaps particularly common in the field of bioethics. A famous example from the US is the National Commission for the Protection of Human Subjects of Biomedical and Behavioral Research, which published the very influential Belmont report in 1978. A more recent example from Scandinavia is the Expert Group in Ethics and Priority Setting, created by the Norwegian government to give advice as to which groups should be given priority for vaccination against the corona virus (Feiring et al. 2020).

One way to accomodate the standard view so as to allow for the use of moral experts, is to say with Peter Singer (1988) that using moral experts is unproblematic as long as politicians only use the expert advice as input, based on which they independently make up their own minds as to what they should do. By contrast, on this view, it would be problematic if politicians deferred to the moral experts, i.e. if they followed their advice not because of the quality of the arguments given, but because it was given by moral experts. In this article, I will suggest, however, that because moral argumentation can sometimes be so complex that non-experts are not able to assess its quality on their own, politicians sometimes will have to defer to moral experts, if they wish to rely on the input of moral experts at all. It therefore becomes crucial to consider whether it can be acceptable for politicians to defer to moral experts. The article will, then, discuss two main questions:

1. Is it the case that politicians sometimes need to defer to moral experts, if they are to use the input of moral experts at all?
2. Is it morally acceptable and democratically legitimate for politicians to defer to moral experts in policy issues?

In discussing these questions, I will proceed as follows. First, I will discuss some general worries as to the very existence of moral experts and the possibility of identifying them. Next, I argue that if politicians are to use expert advice at all, they may sometimes need to defer to moral experts, because of the complexity of the experts’ moral argumentation. I then argue that moral deference might be wrong, as there is a tension between moral deference and democratic legitimacy. Finally, I discuss two ways of arguing that deference to moral experts is not necessary after all. The first, which I criticise, consists in saying that the role of moral experts is only to give conditional advice and say what follows from a given set of values. The second, which I will endorse, consists in saying that since political decisions are subject to a public reason constraint, it would not be acceptable to base policy decisions on moral arguments that are so complex that non-experts cannot assess them.^3^ For this reason, there is no need for politicians to defer to complex moral arguments.

## Some Preliminary Worries About Moral Expertise

As I will use the term, a moral expert is someone who has a greater probability than a non-expert of getting it right in moral matters, or, in other words, of identifying the moral facts.^4^ Given this definition, one might argue that the whole discussion is a non-starter, either because there is no such thing as moral expertise, or because one cannot identify the moral experts. While these objections raise large meta-ethical questions that fall outside of the scope of this article, and are also well discussed elsewhere (e.g. Jones and Schroeter 2012), a few words need to be said about them.

There are two possible reasons why there is no such thing as moral expertise. The first would be that there are no moral facts, so there is nothing for the purported moral experts to be experts about (cf. Jones and Schroeter 2012). This question obviously cannot be settled here, so I will just content myself with a few remarks. First, talk of moral expertise does not commit us to accepting any strong form of moral realism; all it requires is the supposition that moral claims can be mistaken and that some moral claims are more sound than others. And as Jones and Schroeter point out in their discussion of this objection, most meta-ethical theories allow for talking about moral truth in some sense (Jones and Schroeter 2012, p. 220). We can add that the claim that one cannot be mistaken about moral claims would fail to make sense of what is going on in policy debates involving ethical issues, where the participants typically see themselves as discussing what is actually right or wrong (cf. Ebeling 2017, p. 297.) From the point of view of participants in those debates, it thus makes little sense to reject claims of moral expertise on the grounds that there are no moral facts.

A different source of scepticism, however, would be that while there are moral facts, there are no moral experts, because all adult normally functioning human beings are equally capable when it comes to identifying the moral facts. Given our definition of a moral expert as one who has greater probability than others of getting it right in moral matters, we can conclude that there are no moral experts. This claim is implausible, however. As Peter Singer has notably argued, it seems plausible that those who are trained in developing and analysing moral arguments, and have the time to spend on discussing moral questions, will have a better chance of reaching sound moral conclusions (Singer 1972). If this view of moral expertise seems overly theoretical, we might add that those who have had long practical experience dealing with moral dilemmas in a given domain may also tend to get it right more often than others will. Thus medical doctors will probably have a greater degree of expertise in medical ethics than laypersons, all things being equal.^5^ Other non-intellectual skills, such as empathy or imagination, might also be sources of moral expertise. There are thus a number of traits, from intellectual abilities to practical experience, which someone may possess to a larger degree than others and which make it plausible to suppose that they will be moral experts, at least within a certain domain.

A more difficult objection concerns the possibility of identifying the moral experts whose advice one should listen to in cases of disagreement. If two putative moral experts, who are equally well qualified in the sense that they possess to the same degree the traits we think make people moral experts (training in ethics, practical experience, empathy, etc.), disagree on a given issue, the politician needs to know which of them to take advice from. While this problem of knowing which expert to trust is also present when it comes to non-moral experts (Goldman 2001), it is more acute for moral expertise (McGrath 2011). The reason is that for many forms of technical expertise, we have independent access to the facts that purported experts give advice about, and so we can assess which purported experts reach the correct conclusion more often than others do. Indeed, Alvin Goldman describes ‘the use of putative experts’ past track records’ as ‘the best source of evidence’ for choosing between experts (Goldman 2001, p. 106). To use an example from Sarah McGrath (2011): if two weather forecasters make different predictions as to whether it will rain tomorrow, I can check their past track record when it comes to weather prediction in order to know which forecaster to listen to. By contrast, this solution is not available in the case of moral expertise: we have no independent access to the track record of moral experts, as their advice cannot be shown to be correct or not by looking at what happens in the world.

One reply to this difficulty is to note that while there is often disagreement among moral experts, this is not always the case, so at least in cases of non-disagreement, it is possible to listen to moral experts (for a critical discussion of this point, see Goldman 2001, pp. 97–104). Another reply is that sometimes the track record approach *is* available. In the cases when one has had the time to consider a moral question carefully, and where one is convinced that one has found the right solution, one might identify the moral experts by asking which of the putative experts have reached the same conclusion as oneself (see Enoch 2014, pp. 230–233). An objection to this reply is that it makes the use of experts superfluous, since it presupposes that the non-expert *can* reach the right answer. However, the non-expert might not always have the available time to reach the right answer and might for that reason need to appeal to experts. Finally, we should note that while, according to Goldman, track record is the best source of evidence, there are also other sources of evidence that one might apply, such as argumentative skill, and considerations of interests and biases (Goldman 2001; see also Holst and Molander 2017, pp. 237–239).

While none of these replies might be fully satisfactory, we should note that the problem of identifying experts is not unique to moral expertise, and that even the gold standard of past track record is not always easy to apply for technical experts either, when the cases are more complex than that of weather prediction. Thus for example, if an expert predicts that a certain policy will reduce unemployment and unemployment is actually reduced after the policy is introduced, it is not necessarily the case that the predicted outcome followed from the policy that was introduced.^6^ (Cf. Douglas 2008, p. 2.) Despite these challenges in identifying technical experts, the use of such experts is both widespread and unavoidable. Similarly, one reason why it makes sense to discuss which use should be made of moral expertise even in the absence of a full account of how to identify moral experts, is that as a matter of fact, moral experts *do* play a role in many policy-making processes, and so we need to ask how their advice can be used.

## Two Ways of Using Ethical Advice and the need for Deference

Assuming now that there is such a thing as moral experts, and that politicians have a way of identifying the moral experts, what may be the problem with politicians relying on the advice of moral experts when making decisions? The kind of scenario I am considering is one where a politician has to make a decision involving moral issues—either concerning which policy to vote for if he is a member of the legislative (for example whether one should revise the legislation in order to permit research on embryonic stem cells), or which policy to implement if the politician is a member of the executive with delegated authority to make policy decisions (for example how one should prioritise access to vaccines during a pandemic). The politician is unsure as to what is morally right, and therefore asks a moral expert for advice. The context for our discussion is thus one where the politician thinks he will have a higher probability of making the right choice by asking a moral expert for advice than by making the decision on his own.

Crucially, there are two very different ways in which the politician may use the expert advice. (For this distinction, see Hills 2009, pp. 122–123; see also McGrath 2009, p. 322.) I will assume that the advice given by the expert will include both a conclusion as to what the right thing to do is and an argument for this conclusion. In the first way of using expert advice, one uses the expert’s *arguments* as input to one’s own moral reasoning, but one assesses them independently and only follows the advice if one finds the arguments sound. As Hills puts it, you ‘treat the testimony as moral advice, which you subject to critical scrutiny, and you decide whether or not to accept, on its own merits. You take into account what others have said to you as a guide to your own reflections’ (p. 123). In the second way of using expert advice, you simply defer to the expert and follow the advice (at least in part^7^) because the expert gave the advice and not because of the quality of the arguments for the conclusion. In Hills’s words: ‘you simply believe what is said to you. You make no attempt to gather the reasons why *p* and draw conclusions yourself or to devise explanations for moral propositions that you have accepted’ (p. 122). The latter is typically referred to as ‘moral deference’.

The first way of using expert advice is normally seen as unproblematic, also in the case of moral experts. All it involves is the politician being better informed about possible relevant arguments before making a decision; the moral expert does not have any direct influence on the decision as such.^8^ By contrast, if the politician deferred to the expert, the expert would be given a greater opportunity for influence on policy than other citizens, something that may be seen as problematic, as reflected in what I have called *the standard view*. Indeed, a further elaboration of the standard view would be that politicians may use the advice of moral experts as input to their own reasoning, but should not defer to moral experts (cf. e.g. Holst and Molander 2019, pp. 551–552).

### The Complexity of Moral Arguments and the need for Deference

I wish to suggest, however, that sometimes politicians need to defer to moral experts if they are to use the expert’s advice at all. Indeed, when the arguments given for the conclusion are so complex that the politician is not capable of assessing them on his own, he has the choice of either deferring to the expert or not following the advice at all. This is the normal situation when it comes to the use of technical expertise. When nuclear scientists give input as to the safety of nuclear plants, or climate scientists give input as to the probable effects of various measures for mitigating climate change, politicians will rarely have the competence necessary to assess whether the arguments underlying these experts’ conclusions are correct or not—they will just have to trust the experts. If this is seen as less problematic than deference to moral experts, it is, again, because the input given by technical experts is only factual input^9^—nothing follows directly from this input as to what the politicians should do.

*If* it is correct that sometimes (when the arguments behind the moral advice are too complex for the politicians to assess) politicians have the choice between moral deference and not taking advice from the moral experts at all, it seems to follow from the view that moral deference is morally problematic that they should choose the latter option. This comes at a cost, however. Indeed, the reason why politicians consulted the moral expert in the first place, was that they thought that the moral expert has a better chance than themselves of reaching the right conclusion. If they therefore choose not to defer to the expert, they decrease the probability of making the right decision. And, it can be argued, politicians (like all of us) have a duty to do what they can to ensure that they make the right decision (this argument, and the dilemma which follows from it, builds on Enoch 2014). If deference is morally problematic then, politicians are faced with a dilemma: either defer to experts, or decrease their chances of making the right decision.

One way out of the dilemma is to reject the claim that deference is problematic—this is (broadly) the solution chosen by Enoch (2014). But we might also reject the premise of the dilemma, viz. that arguments for moral conclusions are sometimes so complex that they cannot be followed by non-experts. In the rest of this section, I will discuss whether this premise—let us call it the ‘complexity claim’—is correct or not.

### Are Moral Arguments Sometimes too Complex to Understand?

Answering this question in part requires empirical investigation, to see if the relevant target group of expert advice (politicians) understand the arguments behind the advice of moral experts. The question is not purely empirical, however. The reason is that what is relevant is *unavoidable* complexity. If the argument for a conclusion could be made understandable if the expert in question wrote (or spoke) more clearly, the dilemma can be avoided. The question then, is whether the arguments for moral conclusions are sometimes by necessity so complex (if the argumentation is to be sound) that they cannot be understood by non-experts, and that is not a purely empirical question.

I will give two arguments in support of the complexity claim. One is that moral philosophy, like all domains of philosophy, has become increasingly specialised, and the arguments often involve complex technical concepts and theories. As Robert Nozick writes:

Until recently, questions about rationality had been the common possession of human kind, sometimes discussed in intricate trains of thought […] but, nevertheless, largely accessible to intelligent people willing to make the effort. […]

Now things are different—and not just with the topic of rationality. The most fruitful and interesting lines of inquiry about many topics of fundamental human concern have taken an increasingly technical turn. It is impossible now to discuss these topics adequately without a grasp of these technical developments. (Nozick 1993, p. xiv)

A similar point has notably been made by Christopher Bertram (1997), who argues that it follows both from variations in intelligence and from the academic division of labour that political philosophy will develop theories which are too complex for many people to understand. As examples of such theories, Bertram mentions the use of ‘technical issues in decision theory’ for deciding which is the correct decision rule in Rawls’s original position and the role of 'highly technical and controversial moves in bargaining theory' in Gauthier’s discussion of distributive justice (p. 576–577). (I return below to the implications Bertram draws from this fact.)

Against this line of argument, it might be objected that while moral philosophy books might be too complicated for laypeople, the arguments given in the reports produced by moral experts are typically less complex. The reason is that it is part of the expert role to ‘translate’ complex arguments into simpler proposals which are accessible by the relevant decision-makers (Gundersen 2018, p. 56). However, as long as the arguments in these reports build on concepts and theories developed in complicated philosophy books (as will often be the case), a proper assessment of the arguments in such reports requires an understanding of these underlying concepts and theories. The translation might succeed in getting the gist of an argument across, but will often not give the recipient sufficient grounds for assessing the validity of the argument. (Indeed, if the simplified version of the argument were sufficient for assessing the validity of the argument, there would be no need for developing the complex version of the argument in the first place. While this may sometimes be the case, it seems implausible that all the complexity in books in ethics and political philosophy is superfluous.)

A second argument for the complexity claim is that sometimes moral advice will be about issues which are also empirically complex, and a full understanding of the moral argumentation will also require a good understanding of the relevant empirical questions, something politicians will often lack. This will notably be the case for many issues in bioethics, where understanding the moral arguments requires understanding of complex issues in genetics or cellular biology. Advice on such questions is often given by multidisciplinary committees, precisely because of this complexity, and it is implausible that individual politicians should have the competence to fully assess this advice.

In support of both of these arguments, we may note that complexity is relative to the time and resources available to the recipient. It may well be the case that even if a given report produced by an ethics expert would not be too complex for a politician to understand if the politician had six months to read up on the relevant background literature and think about the report, the report *is* too complex to understand in the limited amount of time available to the politician before having to make a decision. The time constraint here is not just a result of some decisions being urgent, such as is the case in a crisis situation, but of the fact that politicians need to make decisions on a large number of different issues, so that the time to be spent on each individual issue is limited.^10^

Giving uncontroversial examples of moral advice in policy reports that is too complex for politicians to understand is not an easy task. Most readers of this article will probably themselves be moral or political philosophers and so their assessment of whether a typical piece of expert advice is too complex to understand would not be representative for the main target audience of such advice. Indeed, when a professional philosopher assesses the complexity of a report there is a twofold risk, of, on the one hand, arrogance in supposing that others cannot understand certain arguments which one understands oneself, and on the other hand, exaggerated humility in underestimating the difficulty of what one has learned over the course of many years of work and study. As I noted above, ultimately it is partially an empirical question whether an argument is too complex for the relevant target group, and this empirical question is not best addressed by the introspection of professional academics. With this caveat in mind, one possible example of too-complex arguments might be some of the reports of the Nuffield Council on Bioethics in the UK.^11^ Indeed, in this case, there is also some empirical evidence that at least some members of the target audience do find the Council’s reports too difficult, in the form of an evaluation commissioned by the Council from a consulting firm (Firetail 2015, pp. 15, 25, 32).

Of course, despite the arguments given above, it may also often be the case that the arguments of moral experts *are* fully understandable by politicians, and in those cases, the dilemma sketched above does not obtain. My argument in what follows is thus limited in scope to the cases where the complexity claim is true.

## The Tension Between Moral Deference and Democratic Legitimacy

I have argued that when the complexity claim is true, politicians will be faced with the choice between moral deference—which many believe is wrong—and having a lower probability of making the right decision. In order to know what they should do in this dilemma, we need to look at why moral deference may be wrong. While there exists different types of arguments against moral deference,^12^ the most salient for our discussion is that moral deference by politicians would decrease the democratic legitimacy of political decisions.^13^

While several reasons may be adduced why there is a tension between giving experts a role in policy development and democratic ideals, I will here focus on one reason that I take to be particularly relevant for moral expertise, viz. that politicians’ deferring to moral experts would give some citizens (the moral experts) a greater chance to influence the outcome than other citizens have. Since it is a basic democratic ideal that all citizens have an equal opportunity to influence political decisions (see Kolodny 2014), deferring to moral experts is in tension with ideals of democratic equality.^14^

Two objections can be raised against the view that deference to moral experts is problematic from a democratic point of view. The first is that as long as the advice of experts is not *binding* on politicians, the decisions based on expert advice remain legitimate. This is the argument behind Peter Singer’s defence of the use of moral experts in the political realm:

Elected leaders in all major nations listen to advice about what they ought to do before they make up their minds, advice not just from their constituents but also from their own appointed advisors. If this is not undemocratic, why should it be undemocratic for elected leaders to seek the advice of a body of people who have more expertise in ethics than they themselves have?

It is important that it is advice we are talking about here, and not binding pronouncements. […E]lected leaders are free to reject the advice of their commission, even of those commissions with ethical expertise. (Singer 1988, p. 155)

Singer’s insistence on the need for the advice to be non-binding can be explained by reference to the democratic desideratum that all citizens have an equal chance of influencing the outcome: indeed, if the experts are not elected, allowing their pronouncements to be binding would grant *some* unelected citizens much more influence than others have. While Singer concludes in favour of the use of moral experts, his contrast between a case where the pronouncements of the moral experts would be binding and the case where politicians are free to reject the advice of moral experts is too simplistic. It overlooks the case where politicians do not understand the argument for the experts’ conclusion. In such a case, the politicians are still free, of course, to reject the advice. However, they do so at the cost of running a great risk of making the wrong—decision—given the assumption that moral experts have a greater probability than non-experts of reaching the right decision. A politician who cares about making the right decision may therefore be committed to defer (at least to some degree^15^) to the moral expert, and this commitment does give the moral expert greater influence than other citizens, since the very fact that the expert says ‘Vote P’ will have a larger weight in the politician’s decision to vote P than the same advice given by an average citizen would have.

A second objection is that my argument over-generalises, since it will also apply to deference to technical experts, and that it is absurd to claim that politicians should not defer to technical experts. Indeed, it is often pointed out that making good policy decisions depends on highly complex empirical evidence, which politicians cannot be expected to assess for themselves; if we thus reject the use of deference, our policy decisions will clearly be sub-optimal (see for example Donahue 2020, pp. 390–391; Holst and Molander 2017; Bertram 1997 p. 578). While I grant that it would be a *reductio* of my argument if it meant that deference to technical expertise was illegitimate, I do not believe that this conclusion follows. The reason is that the questions on which technical experts are asked to give input are not the kind of questions that are the subject of democratic decision-making. Imagine that politicians seek to decide whether to build a new bridge, which will reduce traffic congestion. They may well ask technical experts for input on the following question: ‘What is the probability that the bridge will collapse?’ Once given an answer to that question, they then face a moral question: ‘Given that there is a probability that the bridge will collapse over the next 50 years, how should we weigh the benefit to commuters against the possibility of a serious accident?’ While the answers to both questions are relevant for making the decision whether to build a bridge or not, the first question, being a scientific question, is not the kind of question that one would expect to be decided by a democratic process, whereas the latter, being a moral question, is. Thus it is not problematic from a democratic point of view if technical experts have a greater influence than the average citizen on the technical premises of the decision whether to build a bridge or not, but it is problematic if moral experts were given a greater opportunity to influence the normative premise on which the decision builds (see Christiano 2012, p. 34).

To conclude: political decisions which are the result of politicians’ deference to moral experts will, for that reason, to some degree be lacking in legitimacy even if they have a greater chance of being the right decision than what would be the case if the politician did not defer to experts. We are therefore faced with a dilemma.

## Why Moral Deference may not be Necessary

In this section, I will discuss two ways of avoiding this dilemma, by saying that moral deference is not necessary after all.

### Moral Experts Only Give Conditional Advice

A first approach is to say that the role of moral experts is not to give unconditional advice as to what should be done. Rather, the task of experts is to give conditional advice as to what should be done if one has adopted a certain set of values, or a certain moral theory (cf. Gundersen 2018, p. 57). This way of viewing the role of moral experts is perhaps particularly common in bioethics; thus the sociologist John Evans, in his work on bioethics, writes:

For now I will define *bioethicists* as professionals who use methods in a system of abstract knowledge wherein ethical recommendations are *not* based on their own personal values or the values of a particular group in society, but based on the values of either the individuals involved with an ethical decision or the values of the entire public. (Evans 2011, p. xxi^16^)

Even with this approach, it might be the case that the reasoning as to why, if one has adopted a set of values V, one should do P, is quite complex, so that the politician has to defer to the expert on that issue. But, one might argue, this deference is less problematic than deference to unconditional advice, as the politician does not defer when it comes to the ultimate values which provide the basis for the decision. Following this kind of conditional advice would be more akin to following the instrumental advice of a technical expert, of the form: ‘if you wish to reduce carbon dioxide emissions, you should do X’.

This reply to the dilemma has two problems. First, it underestimates the degree in which a given set of values V underdetermines whether to do P or not. (For a similar point—about how moral theories under-determine specific ethical choices—see Kymlicka 1993, pp. 6–8.) It is simply not the case that once one has identified a set of values V, it is just a technical exercise to see which decision follows on a given issue, for two reasons: first, the values may be quite vague and need to be specified, something which requires substantial moral argumentation. Next, as long as there are several values in the same value-set, there will often be situations where a decision will involve trade-offs between these values, and such a trade-off too requires substantial moral argument.^17^ Both points can be illustrated by Norway’s biotechnology act, the first paragraph of which reads:

The purpose of this Act is to ensure that the application of biotechnology in medicine is utilized in the best interests of human beings in a society where everyone plays a role and is fully valued. This shall take place in accordance with the principles of respect for human dignity, human rights and personal integrity and without discrimination on the basis of genetic background, on the basis of ethical norms relating to our western cultural heritage. (Act of 5 December 2003 No. 100 relating to the application of biotechnology in human medicine, etc, Section 1-1)

It should be obvious that figuring out which conclusions follow from these values on the many controversial issues covered by this Act, such as prenatal diagnostics, stem cell research and gene therapy, requires substantive and often quite complex moral argumentation. If a politician justified his deference to a moral expert on this score by saying that all he asked the expert to do was say what follows from the values he, the politician, already accepts (viz. those listed in section 1-1 of the Act), and that even if he does not understand the expert’s argumentation, this is only a case of technical deference, not moral deference, we would find him disingenuous.

The second problem with this reply, is that it relies on a flawed understanding of moral reasoning as a unidirectional application of a moral theory to a concrete case. In actual practice, when one applies a theory to a given issue, one may well end up revising one’s theory as a result, because the consideration of the concrete case leads one to realise the weaknesses in the position with which one started out.^18^ If a politician thus asks a moral expert to say what follows from a set of values V, but forbids him to make any revisions to this set of values in the process, he may ask for something which is not compatible with sound methods of moral reasoning.

For both of these reasons, the ‘conditional advice’-approach fails.

### Policy Decisions are Subject to an Accessibility Requirement

A more promising way of arguing that moral deference is not required takes as its point of departure the view that political decisions are subject to a requirement of public reason. This requirement entails that the politician must be able to give a justification for his decision that all citizens can see as, in some sense, a proper justification. Exactly what it takes for a justification to satisfy the requirements of public reason is a subject of extensive debate. For my purposes, however, I can remain agnostic as to large parts of this debate. All I require is the relatively minimal^19^ requirement that the justification offered be *understandable* by all citizens, often described as the ‘accessibility’ requirement. This requirement is compatible with public reason also requiring more, such that the justification is acceptable by all citizens, but I take no stand on this issue for the purpose of this article.

I build here on Cécile Laborde’s discussion of the accessibility requirement in her book *Liberalism’s Religion* (2017). Laborde stresses how the accessibility requirement is a precondition for democratic debate: ‘Accessibility articulates what citizens need to share, in particular societies, in order for public deliberation to be possible at all’ (p. 121). I follow Laborde in seeing understandability as central to accessibility.^20^ As Laborde writes: ‘It is one thing to be coerced in the name of reasons one does not understand (such as that life is a gift of God) and quite another to be coerced in the name of reasons that one does not agree with but can engage with […]’ (Laborde 2017, p. 122). While one may require more according to some views of public reason, the case for seeing accessibility as at least a minimum requirement seems strong.

While Laborde primarily discusses the accessibility requirement in relation to religious arguments, it also excludes arguments that for other reasons are not understandable by all citizens, and I take it that one such reason could be that the arguments are too complex.^21^ Indeed, Christopher Bertram has appealed to an ideal of accessibility in order to exclude overly complex arguments from the justification of ‘the terms of association which govern the collective life of citizens’ (Bertram 1997, p. 577).

The relevance of the accessibility requirement, understood as excluding complexity, for the question of moral deference to experts should be clear. If the politician making the decision cannot understand the argument made by the moral expert, there is reason to believe that other citizens will also fail to understand it. And in that case, the argument would anyway be unfit as a justification of a political decision, so there is no need for the politician to defer to the expert on this score. On this view, then, the advice of moral experts can only be used as input for political decisions when the politician understands them, not because moral deference by the politician would be wrong in itself, but because it would be wrong to base political decisions on arguments citizens cannot understand and because there is good reason to believe that if the politician fails to understand the argument, so will other citizens.

To be sure, objections may be raised against this solution too. One concerns the *scope* of the accessibility requirement. Indeed, Bertram limits its scope to the justification of constitutional essentials, in contrast with my suggestion that the accessibility requirement should govern all policy questions on which experts may give advice. It might be argued that by imposing an accessibility requirement that excludes complex arguments on all policy questions, the quality of political decisions risks becoming too low. This seems to be the view of Jeffrey Howard, who, while endorsing an intelligibility requirement on work in ethics and public policy, writes: ‘The insistence that work in EPP [ethics and public policy] be intelligible is distinct from the suggestion that work in EPP be dumbed down, or shorn of its sophistication’ (Howard 2018, p. 29). Howard argues that even if we accept Bertram’s view, ‘it could not plausibly apply to reasoning about specific public policies, which often require a sophisticated combination of empirical and normative principles. […] If the moral truth on some policy question turns out to be complicated […], the fact that it will take extraordinary care and effort to explain that truth properly (and that some people will need to rely on expert testimony to accept the relevant empirical premises) is simply our fate’ (Howard 2018, p. 29).

As I understand them, Bertram’s and Howard’s unwillingness to extend the acceptability requirement to policy decisions seems to be based on the role that complex empirical arguments will play in justifying policy decisions.^22^ Indeed, the examples Bertram gives of the use of experts in policy decisions are all of technical experts (Bertram 1997, p. 578). Likewise, Howard refers to the possibility that *empirical* premises might need to be accepted on testimony, while he does not make a similar point about normative premises. And as noted above, it would indeed be problematic if we did not accept deference in technical matters. However, to the degree that the empirical and the normative premises of a policy decision can be separated, the necessity of citizens deferring to experts when it comes to empirical premises does not entail that they should defer to experts when it comes to normative premises.^23^

A second possible objection is that the politician might justify his decision not by reference to the expert’s arguments, which are too complex to pass the accessibility requirement, but simply by reference to the fact that he followed the advice of a moral expert, who was more likely than him to get it right. Indeed, Sean Donahue has argued not only that relying on testimony when justifying policies is necessary because of our ‘limited resources of reasoning power and time’ (Donahue 2020, p. 190), which bars us from understanding the justifications of all relevant policies, but also that public justification can be compatible with testimony. However, Donahue argues that accepting testimony requires a certain level of trust, and this may not necessarily exist in a given society. Furthermore, this trust is to be established on the basis of a ‘testimonial track record’ (p. 389) and arguably such a track record is harder to establish for moral questions than for empirical questions, for reasons discussed above. (It is notable that Donahue’s examples of necessary reliance on expert testimony (p. 390) all concern empirical matters.)

A further challenge is that if we accept the acceptability requirement, we must settle whether we should require that an argument be understandable by *all* citizens, in which case we might only accept quite simple arguments, and if not, what level of understanding we should use for deciding when an argument is too complex to be accessible. (For discussions, see Bertram 1997 , pp.575 and Lægaard 2020, pp. 14–15.) This is not an objection per se, but it is an issue that needs further development if the accessibility approach is adopted. Indeed, as these objections show, more work is surely required in order to develop the view of the accessibility requirement as excluding overly complex moral arguments, but I hope I have said enough here to make the view seem plausible.

## Conclusion

My argument can be summarised as follows:

1. By definition, a moral expert will have a greater probability of getting it right in moral matters than a non-expert.
2. In some cases the arguments of moral experts are so complex that politicians cannot assess the quality of their arguments. If they are to use the expert advice at all, they have to defer to the experts.
3. It is problematic from a democratic point of view that politicians defer to moral experts because politicians thereby give some citizens more influence than others on political decisions.
4. It follows from 1 and 2 that when the arguments of moral experts are unavoidably so complicated that non-experts do not understand them, policy-makers have a choice between a) not listening to the experts and b) deferring to the experts. Either option has a cost: a) involves a higher risk of making the wrong decision, and b) goes against the democratic desideratum that all citizens have an equal chance of influencing political decisions.
5. This dilemma cannot be avoided by saying that moral experts should only give advice as to which decisions follow from the values the politicians already hold, because even in that case, deference might be necessary.
6. By contrast, the dilemma can be avoided if we impose a requirement that policy decisions must be based on reasons accessible to all citizens, since this accessibility requirement excludes policies building on too-complicated arguments.

An apparent cost of the solution I have proposed, however, is that in some cases, experts will be barred from giving the best advice they can give and policy-makers will miss out on moral knowledge, thus making worse decisions than they otherwise would have done. It might therefore be claimed that the proposed solution simply repeats the dilemma sketched above in a somewhat different form: either a) not listen to experts and therefore accept a higher risk of making the wrong decision or b) disrespect the accessibility requirement.^24^ The objection can be met, however, if we reject the view that the accessibility requirement forces us to accept a ‘second-best’ moral solution, in the cases where there is no accessible argument for the best moral solution. On the contrary, accessibility is a necessary criterion for a solution being the best, so that a solution for which no accessible justification exists cannot be seen as the best solution. The reason is that accessibility is a requirement for a solution being (in one sense or another) justifiable to all and in a ‘democratic community’ (to use Bertram’s term) a solution that is not justifiable to all cannot count as the best solution. (For this point, see Bertram 1997, pp. 574–575.) If we accept this line of argument, policy-makers can refrain from deferring to moral experts without thereby running the risk of opting for sub-optimal moral decisions.

## Footnotes

1 It may be objected that even factual premises cannot be guaranteed to be morally neutral, as the values held by the scientific experts influence their factual findings (see Kitcher 2011, pp. 31–40). While there is some validity to this objection, its weight should not be exaggerated, as it will often be possible to separate an expert’s technical claims from his value claims (see Holst & Molander 2017, p. 240).

2 Peter Singer formulates the standard view in a similar way, though he rejects the view thus understood (Singer 1988, p. 155). For another statement of the standard view, see Vibert 2007: ‘What underlies the new separation of powers is a distinction between the empirical component of public policy and the value judgements. The making of public policy involves both elements—the factual evidence and the social or political judgements to be made in the light of that evidence. Unelected bodies have an advantage in dealing with the empirical components of public policy and elected bodies in choosing the values to be reflected in public policy’(p. 2). (See also Christiano 2012.)

3 The argument defended in this article in several ways resembles the argument made by Christopher Bertram (1997), who argues that because of the complexity of certain arguments in political philosophy, these arguments should be excluded from the justification of ‘the terms of association which govern the collective life of citizens’ (p. 577). Bertram’s main focus, however, is on the arguments justifying constitutional essentials, and not on the role of moral experts in giving advice to politicians concerning more day-to-day policy questions (See below).

4 Compare Alvin Goldman: ‘As a first pass, experts in a given domain […] have more beliefs (or high degrees of belief) in true propositions and/or fewer beliefs in false propositions within that domain than most people do (or better: than the vast majority of people do)’(Goldman 2001, p. 91). Goldman goes on to nuance his definition in various ways, but for my purposes in this article, what is essential to the expert role is the expert’s greater propensity to get it right.

5 This distinction between theoretical skills and practical experience as a source of expertise is similar to, though not identical with Jones and Schroeter’s distinction between ‘the intellectual model’ and the ‘practical wisdom model’ of moral expertise, Jones and Schroeter 2012, pp. 218–219.

6 I am grateful to Cathrine Holst for pressing this point and for the example.

7 Politicians (or anyone using moral experts) will rarely fully defer to the expert, in the sense that they will follow their advice no matter what the content of the advice is. But as long as they give greater weight to the advice than what the arguments given for the conclusion merit, on the grounds that the advice was given by an expert, this counts as deference to some degree.

8 This point should be nuanced, as the types of arguments presented to the decision-maker may influence the decision made even if the decision-maker makes up her own mind based on her assessment of these arguments.

9 To be sure, often their input will include instrumental advice as to which means are best suited for reaching a certain goal (cf. Gundersen 2018, p. 57). But even for this kind of advice, it is the case that without further normative premises, nothing follows from the advice as to what the politician should do.

10 Sean Donahue makes an analogous point when discussing ‘high-standard’ views of public justification, which requires that citizens can understand the policy justifications given to them, so that they do not have to rely on testimony: while this requirement might be feasible for *one* policy, they will not have the time to understand the justifications given for all the policies of interest to them (Donahue 2020, pp. 383–384).

11 The Nuffield Council is not appointed by the government with an explicit mandate to give advice to political decision-makers, but arguably they serve a similar function as committees created with this explicit mandate. (Cf. Montgomery 2017.) (Admittedly, it might be argued that the fact that the Nuffield Council is not appointed by the government in order to give advice makes its functioning very different from government-appointed expert committees. Whether that is the case or not does not, however, detract from the value of the Nuffield Council’s reports as possible examples of too complex arguments.) (I am grateful to an anonymous referee for stressing the need for examples of reports that may be too complex.)

12 A different type of argument against moral deference is that if one acts out of moral deference, one may outwardly do the right thing, but one does not understand why one’s action is right. As a result, one’s action would lack moral worth (Hills 2009) and one would not count as virtuous, despite doing the right thing. (See also Howell 2014.) While important, I believe such arguments have greater relevance in the domain of personal morality than in political morality. (For a related point, cf. Skarsaune 2016, pp. 354–356.)

13 I take legitimacy to be a question of degrees, rather than an either/or-question. Thus I do not claim that any deference to moral experts necessary makes a decision illegitimate, but that such deference will detract from the legitimacy of a decision and thus be problematic from a democratic point of view.

14 Kolodny notes that it is unproblematic if a person A has a greater opportunity for influence than a person B because the person listening to their arguments find A’s reasons to be more convincing than B’s reasons. Thus if a moral expert has a greater opportunity of influence than a non-expert because the recipient of the advice finds, based on her own autonomous judgement, the reasons underlying the expert’s advice better than the reasons underlying the non-expert’s advice, this is not problematic from a democratic point of view. In Kolodny’s words, ‘while “judgment-independent” inequalities in opportunity for informal influence may be problematic, “judgment-dependent” inequalities—which merely result from the influenced person exercising his judgment—certainly are not’(p. 334). However, that is not the situation we are in when politicians *defer* to the moral expert. In that case, the greater opportunity of influence of the moral expert is *not* judgement-dependent, since the politicians defer to the expert precisely because they cannot understand the expert’s arguments and make up their own mind about the soundness of these arguments. (I am greatful to an anonymous referee for suggesting that I relate my views to Kolodny’s distinction between judgement-dependent and judgement-independent inequalities.)

15 See footnote 7 above for this qualification.

16 By contrast, moral philosophers working on bioethics and who give advice based on their own views are labelled by Evans as ‘rogue bioethicists’ (p. xxv), with the clear implication that they do not operate within the standard framework of expert advice.

17 My arguments here are akin to Alf Ross’s arguments as to why the social scientist cannot view himself as simply a technician implementing political goals (Ross and Holtermann 2013, pp. 409–410). As Ross writes: ‘The political decision therefore always has the nature of a decision, not a solution (as in the solution to a technical problem)’ (Ross and Holtermann 2013, p. 410; my translation).

18 There is a certain parallel between this point and Thomas Nagel’s discussion of the relation between internal and external criticism in Nagel 1986 p. 125.

19 But not fully uncontroversal, cf. Vallier (2011).

20 For this point, see also Nagel 1987. For a different understanding of accessibility, see Vallier (2011).

21 Thomas Nagel also suggests (tentatively) that the accessibility requirement may exclude complex reasoning (Nagel 1987, p. 236).

22 Bertram does provide a separate line of argument, based on the importance that decisions concerning terms of political association be ‘a person’s own choice’, unlike the choice of medical treatment, where one can defer to the doctor. I believe that this argument too could be extended to cover also policy decisions with a clear normative aspect.

23 Indeed, Bertram notes in a footnote that he has received the suggestion ‘that public reasons concerning the normative, as opposed to the empirical, component of the case for all legislation as well as for constitutional essentials must be formulated so as to meet the accessibility requirement’ (Bertram 1997, p. 579). This suggestion, which Bertram neither endorses nor rejects explicitly, is compatible with the view I suggest here.

24 I am grateful to Lars Christie for pressing this point against me.
